# The Insulin/IGF System in Mammalian Sexual Development and Reproduction

**DOI:** 10.3390/ijms20184440

**Published:** 2019-09-09

**Authors:** Yasmine Neirijnck, Marilena D. Papaioannou, Serge Nef

**Affiliations:** Department of Genetic Medicine and Development, University of Geneva, 1211 Geneva, Switzerland

**Keywords:** INS/IGF signaling, sexual development, mammalian reproduction, steroidogenesis, Sertoli cell development

## Abstract

Persistent research over the past few decades has clearly established that the insulin-like family of growth factors, which is composed of insulin and insulin-like growth factors 1 (IGF1) and 2 (IGF2), plays essential roles in sexual development and reproduction of both males and females. Within the male and female reproductive organs, ligands of the family act in an autocrine/paracrine manner, in order to guide different aspects of gonadogenesis, sex determination, sex-specific development or reproductive performance. Although our knowledge has greatly improved over the last years, there are still several facets that remain to be deciphered. In this review, we first briefly outline the principles of sexual development and insulin/IGF signaling, and then present our current knowledge, both in rodents and humans, about the involvement of insulin/IGFs in sexual development and reproductive functions. We conclude by highlighting some interesting remarks and delineating certain unanswered questions that need to be addressed in future studies.

## 1. Introduction

From an evolutionary point of view, metabolism, growth and reproduction are tightly connected. Indeed, fertility reaches its full potential after completion of growth and puberty, and depends on the individual’s metabolic status (for a review, see [1]). Indicative of these intricate connections is the fact that excessive leanness or obesity in both men and women are associated with reproductive dysfunctions and hypogonadotropic hypogonadism [1].

The interconnection of metabolism, growth and reproduction is a direct result of common regulatory networks and signaling pathways [2]. In particular, the insulin-like family of growth factors (henceforth referred to as the insulin/IGF system) plays a pivotal role in the regulation of cell metabolism, growth, proliferation, differentiation and survival, and affects nearly every organ [3]; its constituents are important players in a network of biochemical events that link metabolic pathways, mitogenic processes and reproductive functions.

Remarkably, the insulin/IGF system is characterized by a high level of complexity due to the presence of multiple ligands, receptors and signaling pathways. In addition, the bioactivities of insulin, IGF1 and IGF2 depend on the concerted effects of a number of factors, including nutritional status, developmental stage, ligand biosynthesis, interactions with other hormonal systems, regulation of ligand bioavailability by IGFBPs (IGF binding proteins) and others. Dysregulation of the insulin/IGF axis has major pathological implications, ranging from metabolic disorders to growth deficits, cancer development and reproductive disorders. Importantly, although it has long been known that the reproductive capacity of an individual is regulated by the hypothalamic–pituitary–gonadal (HPG) axis [4], accumulating evidence has gradually revealed that the activity of local gonadal factors such as IGFs is also of prime importance for reproductive performance.

In this review, we focus our attention on the insulin/IGF system, whose extensive study has demonstrated its key roles in mediating several processes of adrenogonadal development and functions in mammals. First, we briefly describe the components of the insulin/IGF system; next, we provide a short overview of gonadogenesis, sex determination, and sex-specific development in males and females; then, we present gene expression and biological data that reveal the roles played by insulin/IGF signaling during sexual development and in mediating reproductive functions. In the final section, we conclude by outlining several questions that remain to be answered, as well as directions that need to be followed in future studies.

## 2. The Insulin/IGF System

**Figure 1:**
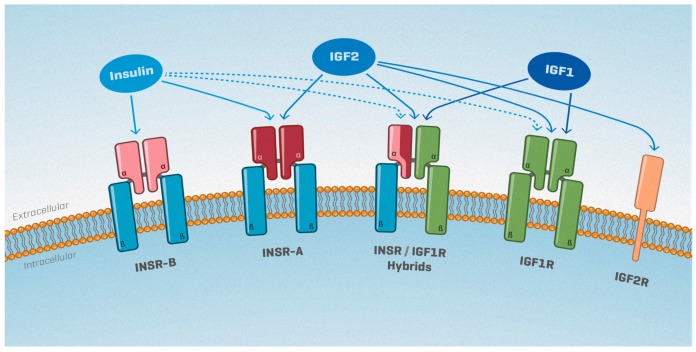
The insulin/insulin-like growth factors (IGF) system: Ligand–receptor binding affinities. Insulin receptor (INSR) and insulin-like growth factor receptor (IGF1R) receptors are each composed of two αβ dimers. *InsR* has two splice variants, whose respective proteins (INSR-A and INSR-B) differ in their extracellular α subunit. INSR αβ dimers can bind to IGF1R αβ dimers, forming INSR/IGFR hybrid receptors. The M6P/IGF2R receptor is unrelated to INSR and IGF1R and lacks intrinsic tyrosine kinase activity. The relative binding affinities of each receptor to insulin, IGF1 and IGF2 are indicated by solid arrows (high affinity) or broken arrows (low affinity).

The insulin/IGF system is composed of three members, including insulin and insulin-like growth factors 1 (IGF1) and 2 (IGF2) (Figure 1). IGFs are small, single-chain, mitogenic polypeptides that are structurally similar to proinsulin [5]. Locally produced IGFs are mainly involved in autocrine/paracrine activities, whereas circulating pancreas-produced insulin and liver-produced IGF1 mediate endocrine activities [6]. The bioavailability of IGF1 and IGF2 is regulated by a family of six high-affinity binding proteins (IGFBP1-6), which sequester IGFs or act as an IGF “reservoir” by increasing their half-life and allowing for their gradual release [7].

IGFs and insulin exert their physiological effects by activating two tyrosine kinase receptors, namely the type-1 insulin-like growth factor receptor (IGF1R) and the insulin receptor (INSR) (for a review, see [8]) (Figure 1). Each receptor is a heterotetrameric glycoprotein composed of two ligand-binding extracellular α subunits and two transmembrane β subunits. In addition, two alternative splicing isoforms of *INSR* exist: the fetal *INSR-A* isoform, which lacks exon 11 and mediates mitogenic effects [9], and the classical *INSR-B* isoform, which predominantly signals insulin’s metabolic activities. Of note, cells that co-express *INSR* and *IGF1R* can also form hybrid receptors composed of the α and β subunits of INSR-A or INSR-B bound to the α and β subunits of IGF1R (Figure 1).

The affinity of INSR and IGF1R towards each IGF differs significantly (Figure 1). Insulin binds with the greatest affinity to both INSR isoforms, but not to the hybrid INSR/IGF1R receptors [9,10]. IGF1 mediates its mitogenic effects primarily through IGF1R, but can also bind IGF1R/INSR-A and IGF1R/INSR-B hybrid receptors [11,12,13,14,15]. Finally, IGF2 signals through IGF1R, but has also been found to bind to the fetal ISNR-A with an affinity close to that of insulin [9]. IGF2 can also bind to the INSR-A/IGF1R hybrid receptor, as well as to the cation-independent mannose-6-phosphate/IGF2 receptor (IGF2R). It should be noted, though, that the M6P/IGF2R is unrelated to INSR and IGF1R, lacks intrinsic tyrosine kinase activity, and is thought to serve as a mechanism for clearing circulating IGF2 [16].

**Figure 2:**
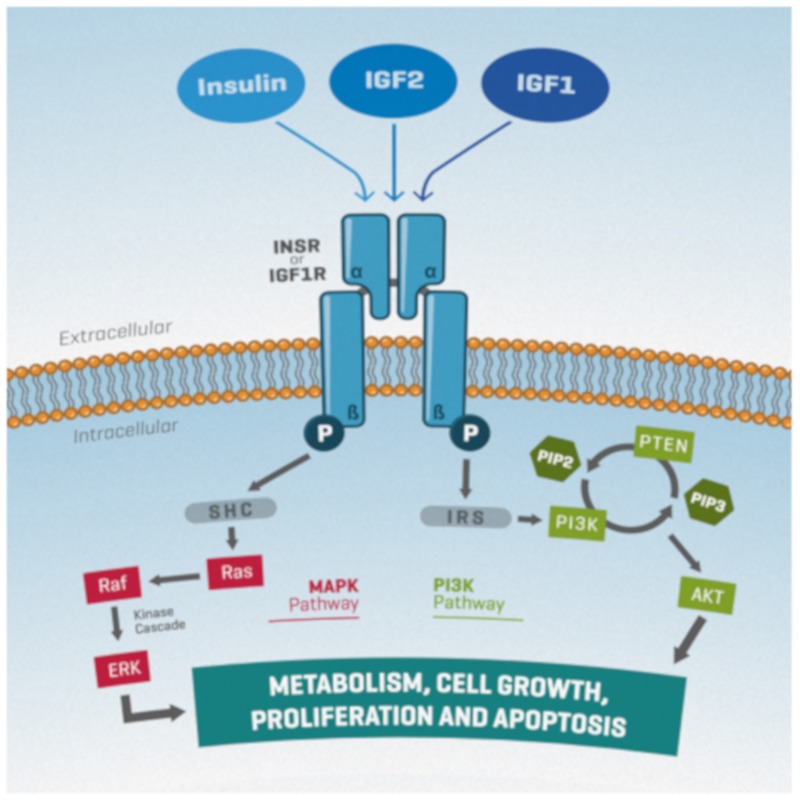
A simplified schematic representation of insulin/IGF signaling. Ligand binding to their receptors leads to autophosphorylation of the β subunits. Subsequent recruitment and phosphorylation of IRS (insulin receptor substrate) or SHC (Src homology domain-containing) proteins leads to the activation of the MAPK (Ras/Raf/ERK) or PI3K (PI3K/PTEN/AKT) signaling pathways. AKT activation is modulated positively and negatively by PI3K and PTEN, respectively, through PIP3 or PIP2 production. Both pathways are associated with cell proliferation, differentiation, metabolism, and survival. Arrows indicate signal transduction.

Upon ligand binding, phosphorylated receptors recruit IRS (insulin receptor substrate) proteins or SHC (Src homology domain-containing) proteins, leading to activation of two main pathways, the phosphatidylinositol-3 kinase (PI3K)/PTEN/AKT and the ERK/MAPK, both of which are associated with proliferation, differentiation, metabolism, and survival [17] (Figure 2). However, evidence has pointed towards the involvement of additional pathways, such as the JAK-STAT [18].

Although several physiological roles of the insulin/IGF system have been revealed over the years, our understanding is still somewhat limited due to the system’s particular complexity. In addition to the multiplicity of ligands, receptors, IGF binding proteins, IGFBP proteases; the variability in ligand binding affinities for their receptors; and the variety of signaling pathways activated downstream of receptor activation, further complexity is added due to the fact that INSR and IGF1R can translocate to the nucleus and function as transcription factors [19].

## 3. Genital Ridge Formation, Sex Determination and Sex-Specific Gonadal Development

In mammals, the establishment of functional testes and ovaries involves three sequential processes: formation of the bipotential gonadal primordium, followed by sex determination and sex-dimorphic differentiation of the gonads. (Importantly, this review is not intended to provide exhaustive information on gonadal development and sex determination; hence, we would like to refer the readers to a number of relevant reviews e.g., [20,21]).

In mice, the gonadal primordium arises as a thickening of the epithelium along the coelomic surface of the mesonephros around embryonic day 10.0 (E10.0). At this stage, the gonadal primordium is composed mostly of primordial germ cells (PGCs) and *Nr5a1*-expressing somatic cell precursors [22]. These multipotent somatic progenitors give rise to the supporting and steroidogenic lineages: Sertoli (SC) and Leydig (LC) cells in the testis, granulosa and theca cells in the ovary.

The first sex dimorphic event, or sex determination, coincides with the commitment of a subset of somatic progenitors to the supporting fate. Mutually antagonistic male and female programs are initiated around E11.5: An *Sry/Sox9/Fgf9* cascade is triggered in XY individuals [23,24,25], whereas an *Rspo1/Wnt4/Ctnnb1/Foxl2* network drives ovarian fate in XX embryos [26]. As a result, somatic precursors adopt a supporting lineage fate and differentiate into pre-SCs in males and pre-granulosa cells in females. Once established, supporting cells direct the sex differentiation of other lineages such as the steroidogenic and germ cells.

Fetal LCs (FLCs) of the testis first appear around E12.5 [27] and are thought to differentiate from several sources, including interstitial cells derived from the coelomic epithelium and migrating cells derived from the mesonephros [28]. FLCs are responsible for masculinizing the male urogenital system through androgen production. However, after birth, they are gradually replaced by adult LCs (ALCs) [27], which produce the testosterone responsible for spermatogenesis, maintenance of secondary sex characteristics and fertility. In females, theca cells become distinguishable after birth upon receiving granulosa cell-derived signals [29], and produce androgens that are further converted into estrogens by the granulosa cells.

Germ cell sex determination is defined by the timing of meiotic entry. XX PGCs differentiate around E12.5–13.5 as meiotic oocytes that arrest at prophase I, whereas XY PGCs develop as mitotically quiescent prospermatogonia around E14.5–16.5 (for comprehensive reviews, see [30,31]). In both sexes, gametogenesis is completed during adulthood. Spermatogenesis, the process through which diploid spermatogonia differentiate into haploid spermatozoa, is a continuous process occurring throughout life; oocyte maturation, on the other hand, occurs at each estrous cycle and oogenesis is fully completed only upon fertilization.

Although gametogenesis during adulthood is under the control of the HPG axis, the somatic environment of the gonad also has a major impact on gamete production. For instance, SCs are in direct physical association with all types of germ cells (GCs) and provide them with structural support and a preserved environment that is tightly regulated by the blood-testis barrier; they also assist their movement towards the seminiferous epithelium lumen and sustain their development through their secretory products (for comprehensive reviews, see [32,33]). Importantly, an individual SC can only support a finite number of GCs; hence, the final testis size, the number of GCs and the adult sperm output are directly linked to the total number of SCs [34]. Similarly, in females, oocytes are enclosed together with granulosa and theca cells in follicles, the functional unit of the ovary; they are in physical contact with granulosa cells, which regulate their growth and maturation [35].

## 4. Expression of Insulin/IGF Family Members in the Gonads of Mice and Humans

IGF1 is synthesized by most, if not all, tissues in the body, where it exerts autocrine or paracrine effects [36]. IGF1 is also produced by the liver and then secreted as an endocrine hormone into the bloodstream. Regulation of hepatic IGF1 production is complex and involves mainly the function of growth hormone (GH). IGF2 is also expressed in numerous tissues, including the liver, but its hepatic production is not regulated by GH. Importantly, the *IGF2/Igf2* gene displays genomic imprinting: in humans, as well as other mammals like rodents, the paternally inherited *IGF2/Igf2* allele is expressed, while the maternal allele is transcriptionally silent [37]. This monoallelic expression results from differential DNA methylation of the two alleles, ensuring correct gene dosage, otherwise leading to several types of growth disorders [37,38]. Sex-specific methylation imprints are initially established during embryonic life, in developing oocytes and prospermatogonia [39]. They are transmitted to the next generation, maintained in somatic cells of the embryo, while erased in PGCs. In mice, *Igf2* is expressed mainly during embryonic development and ceases a few weeks after birth [40]. Thus, IGF2 exerts its proliferative and anti-apoptotic actions only during embryonic and fetal stages, while postnatal growth is IGF2-independent. In humans, however, a different pattern is observed, since both *IGF1* and *IGF2* remain expressed throughout life and have been reported to act as important modulators of muscle growth and differentiation [41].

**Figure 3:**
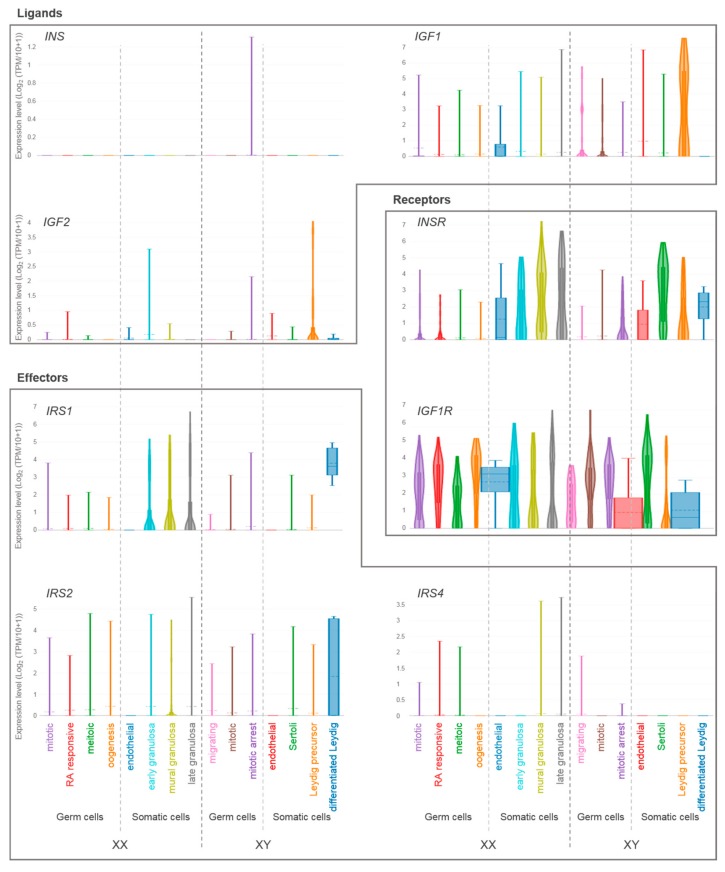
Single-cell gene expression levels of insulin/IGF system members in developing human ovaries and testes. Violin plots showing relative expression levels of genes coding for ligands (*INS, IGF1, IGF2*), receptors (*INSR, IGFR*) and effector proteins (*IRS1, IRS2, IRS4*) in human fetal gonads (data from Li et al., [46], adapted from The ReproGenomics Viewer [47,48]). Somatic and germ cell clusters for each sex are indicated on the x-axis. Expression levels are indicated on the y-axis. TPM, transcripts per million; RA, retinoic acid.

Our group recently provided a detailed expression profile of insulin/IGF system members in murine developing testes using single-cell RNA sequencing [42] (Figure 3 and Table 1): *Igf1* and *Igf2* transcripts were found in all cell types, with notably higher expression levels in interstitial progenitors. In contrast, *Insr* transcripts were found exclusively in LCs, whereas *Igf1r* was expressed in all cell types. The gene coding for the effector protein *Irs2* was found to be expressed at higher levels in immature SCs, whereas *Irs1*, *Ins1,* and *Ins2* transcripts were not detected or minimally expressed. Overall, these data suggest that local testis-secreted IGFs exert their paracrine effects on SCs and LCs.

After birth, *Igf1* and *Igf2* are both robustly expressed in GCs, markedly in spermatogonia [43], while all IRS proteins are found in the adult mouse testis [44]. Notably, although insulin is primarily produced by pancreatic β-cells, it has recently been shown that the testis also produces insulin; interestingly though, it is pancreatic, and not testicular, insulin that regulates the male HPG axis and is thus essential for mouse male fertility [45].

In human developing gonads, *INSR* is preferentially expressed in somatic cells in both sexes, whereas *IGF1R* is ubiquitously expressed (Figure 3 and Table 1, data from [46]). *IRS1* and *IRS2* are highly expressed in LCs in males, and only *IRS1* is expressed in females, specifically in granulosa cells. *IRS3*, *IRS4,* and *INS* are minimally expressed or not detected. Interestingly, *IGF1* and *IGF2* are preferentially expressed in PLCs (progenitor Leydig cells), suggesting that, similar to what we observed in XY mice, locally PLC-produced IGFs exert paracrine actions on surrounding somatic cells.

## 5. Biological Effects of the Insulin/IGF System in Sexual Development and Reproduction

### 5.1. Differential Contribution of IGF Ligands to Sexual Development and Reproduction

The fact that several members of the insulin/IGF system play significant roles in sexual development has been firmly established over the last decades, mainly through the use of in vivo mouse models (summarized in Table 1).

Originally, it was shown that mice with a targeted disruption of the *Igf1* gene exhibit intrauterine growth retardation and postnatal growth failure, ultimately leading to dwarfism, with a ~70% reduction in body weight [50]. Interestingly, both male and female *Igf1* mutant mice are infertile with reduced libido: Males have small testes and reduced sperm count due to defects in androgen synthesis by LCs, while mutant females fail to ovulate despite hCG stimulation, possess an infantile uterus and exhibit hypoplasia of the myometrium. In men, homozygous mutations of the *IGFI* gene result in similar phenotypic features, except for the fact that their reproductive system is not affected [57,58,59,60]. However, IGF1 or GH administration to boys/men with non-GH deficient (GHD) short stature, congenital isolated GHD or GH resistance improves reproductive parameters such as testicular volume and sperm production (reviewed in [62]). This suggests that although *IGF1* mutations have so far been shown to be compatible with normal testicular development and function, exogenous IGF1 administration is able to positively affect testicular parameters.

Contrary to IGF1, no essential roles in male sexual development have been revealed for IGF2. Deletion of *Igf2* results in placental insufficiency [52,63], intrauterine growth restriction [64,65] and reduced fetal weight, but these abnormalities are not accompanied by any testicular defects.

The role of insulin in testis development has been more difficult to decipher. There have been a few publications suggesting a possible link between insulin and testis development in humans and pigs [66,67], but for the time being, this link has not been firmly confirmed. Nonetheless, it has been shown that overexpression of *INS* in LCs reduces the number of germ cells and gradually leads to infertility in mice [56], and that insulin induces the expression of *DAX1* (officially *NR0B1*) in LCs, which in turn inhibits testicular steroidogenesis both in vivo and in vitro [49].

Nevertheless, it should be kept in mind that, because of the redundancy of the IGF ligands, a potential role of IGF2 and insulin in gonadal development and function may be masked by the presence of IGF1.

### 5.2. The Insulin/IGF System and Sex Determination

The multiplicity of interactions and the large range of affinity between IGFs and their cognate receptors, either as homodimers or as hybrid heterodimers, implies that IGF signaling must include both IGF1R and INSR-mediated transduction. In 2003, Nef et al. [53] generated triple knockout mice for *Insr*, *Igf1r*, and *Insrr* (insulin receptor-related receptor, an orphan receptor of the insulin receptor gene family [68]). This study showed that insulin/IGF signaling is absolutely essential for sex determination and testis differentiation in mice, since triple knockout embryos display a complete male-to-female sex reversal, characterized by reduced expression of *Sry* and *Sox9* [53].

Later on, a similar phenotype was recapitulated in mice that were mutant only for *Insr* and *Igf1r*: double mutant embryos show reduced proliferation rates of somatic progenitor cells in both XX and XY gonads, prior to sex determination, due to a reduction in *Nr5a1* expression. As a result, mutant mice display complete agenesis of the adrenal gland and absence of testis development [54]. Notably, a delay in ovarian differentiation and germ cell entry into meiosis is also observed in these mice, suggesting that, regardless of the genetic sex, gonads lacking insulin/IGF signaling remain in an undifferentiated state, with no clear activation of either testicular or ovarian genetic programs for several days. In fact, prior to sex determination, insulin/IGF signaling orchestrates a complex and dynamic transcriptional program in the bipotential somatic precursors, which, when prematurely altered, results in adrenal specification and gonadal development failures.

Overall, these results highlight the essential role played by the insulin/IGF signaling pathway in mediating different aspects of adrenogonadal development, such as adrenal specification, testicular differentiation and ovarian development. Importantly, they have shed light on a crucial, but so far underestimated, signaling pathway underlying sex determination.

Such studies, though, are somewhat limited due to the fact that the constitutive invalidation of *Insr* or *Igf1r* in mice leads to perinatal lethality [53,54]. More recently, the use of animal models with cell-specific disruptions has allowed a detailed investigation of the roles played by individual insulin/IGF signaling members in mediating adrenal, testicular and ovarian development and function (these will be analyzed in subsequent sections).

### 5.3. The Role of the Insulin/IGF System in Sertoli and Granulosa Cells

In both sexes, gamete production critically relies on the supporting lineage. Indeed, as mentioned earlier, the physical association of supporting cells with GCs provides the latter with structural support, as well as with an appropriate somatic environment that regulates spermatogenesis and folliculogenesis—and, hence, fertility.

The biological actions of IGFs on SCs and granulosa cells have been extensively studied both in vitro and in vivo (see below). In vitro, IGFs have been found to be crucial for FSH-mediated events in supporting cells of both sexes. In particular, IGF1/IGF1R function is essential for FSH-mediated AKT activation, subsequent steroidogenic enzyme expression and estradiol production in human and rodent granulosa cells [69]. In a similar manner, IGF1 and FSH synergistically activate AKT/PI3K in immature rat SCs [70]. In this system, IGF1 inhibits FSH-mediated steroidogenic enzyme expression and estrogen production [71]. Thus, although IGF1 triggers the activation of the same signaling molecules in both Sertoli and granulosa cells, this results in opposite biological responses in regard to estrogen production, thereby highlighting once again the complexity of IGF signaling.

The use of animal models with cell-specific gene disruptions has been a tremendous contribution to the deciphering of insulin/IGF activity with respect to spermatogenesis and folliculogenesis. Recently, our studies with SC-specific knockout mouse models for the *Insr* and/or *Igf1r* genes provided in vivo evidence that the insulin/IGF receptor family plays a major role in regulating immature SC proliferation, maturation and, ultimately, daily sperm production [43]. Adult testes of mice lacking both *Insr* and *Igf1r* in SCs (SC-*Insr;Igf1r*) display a 75% reduction in testis size and daily sperm production, as a result of a reduced proliferation rate of immature SCs during the late fetal and early neonatal testicular period. On the other hand, testes lacking only *Insr* or *Igf1r* in Sertoli cells display a weight reduction of 14% and 35% respectively, compared to control littermates. The fact that concomitant ablation of both receptors results in a much more severe reduction in testis weight implies the existence of significant redundancy, but also suggests that INSR and IGF1R act in a synergistic manner to regulate SC number and testis size. In fact, taking into account both the ligand binding affinities and the relative contribution of INSR and IGF1R to the phenotype, we propose that IGF2 exerts the major role in promoting SC proliferation, since, as previously mentioned, IGF2 signals through both the IGF1R and the fetal ISNR-A isoform [9,14]. Moreover, when compared to the influence of known regulators of neonatal SC proliferation such as FSH, androgens or thyroid hormones, our data indicate that, in fact, IGFs are proportionally the major regulators of SC number and testis size in mammals. Indeed, mice lacking the FSH receptor specifically in SCs show a 55–60% reduction in testis weight and SC number [72] (whereas SC-*Insr;Igf1r* mice show 75%). Importantly though, an investigation of the potential interactions between FSH and the insulin/IGF pathway has shown that insulin/IGF signaling is actually necessary to mediate the proliferative effects of FSH on immature SCs [43].

As mentioned in Section 2, upon IGF binding, phosphorylated receptors recruit IRS proteins or SHC proteins, leading to activation of two main pathways, the PI3K/PTEN/AKT and the ERK/MAPK [17]. The relevance of IRS proteins for SC development and sperm production in vivo has been revealed through the constitutive deletion of the genes coding for these proteins: *Irs2* knockout mice (but not *Irs1* mutants) show a 45% testis weight reduction, associated with fewer SCs, GCs, and epididymal sperm, whereas LC number and testosterone production are not affected [44]. Crucially, this study has revealed the importance of IGF/IRS2 signaling in mediating SC development, testis size and subsequent sperm production; however, the cell-autonomous effects of IGFs could not be analyzed in this particular mouse model. In addition, although several reports have indicated that, in vitro, IGF1 signals through the PI3K pathway in supporting cells, the mechanism underlying the in vivo effects of insulin/IGF signaling in promoting SC proliferation, testis size and sperm production was only recently elucidated. More precisely, the generation of mice with a SC-specific deletion of *Pten* (phosphatase and tensin homolog), a negative regulator of the PI3K/AKT pathway, has allowed researchers to decipher the IGF-dependent intracellular events promoting SC proliferation. Although ablation of *Pten* alone appears to be dispensable for SC proliferation and spermatogenesis, inactivation of *Pten* in the absence of *Insr* and *Igf1r* rescues SC proliferation rate during late fetal development, as well as subsequent testis size and sperm production [42]. These findings suggest that, in vivo, IGFs promote the proliferation of immature SCs through the IGF/PTEN/PI3K pathway.

In females, Zhou and colleagues have shown that the in vivo inhibition of IGF1R activity or expression prevents the FSH-mediated granulosa cell functions, such as expression of steroidogenic genes and estradiol production [69]. More precisely, they have found that IGF1R signaling is necessary for FSH-induced activation of AKT, as well as for the subsequent differentiation of human cumulus granulosa cells to the mural/preovulatory stage [73]. In fact, there exists an intricate interplay between IGF2 and FSH during granulosa cell differentiation: FSH is a potent enhancer of IGF2 expression in human granulosa cells and, in its turn, IGF2 activation of IGF1R and AKT is necessary for FSH to stimulate *CYP19A1* expression and proliferation of granulosa cells [74]. Overall, these findings suggest the existence of a positive loop interaction between FSH and IGF2 that is critical for human granulosa cell proliferation and differentiation.

In 2017, the same research group provided the ultimate proof of a crosstalk between IGF and FSH signaling within the ovaries: the authors generated a conditional mouse knockout model in which *Igf1r* is specifically ablated in ovarian granulosa cells, and found that *Igf1r* expression is essential for steroidogenesis, follicle survival and fertility in females. Their study revealed that the ovaries of such mutant mice are smaller than those of control littermates, contain no antral follicles even after gonadotropin stimulation, and are therefore infertile [75]. More specifically, the authors found that the knockdown in *Igf1r* expression causes a loss of responsiveness to FSH in preantral follicles, and thereby concluded that IGF1R is indeed critical for FSH-induced differentiation of granulosa cells in vivo.

Overall, a plethora of studies performed during the last few decades has thoroughly characterized the relevance of insulin/IGF signaling in the development of supporting cells and reproduction on the whole. In both sexes, IGFs signal through the PI3K/PTEN/AKT pathway and are required for FSH-induced cellular processes. However, the biological responses upon FSH/IGF signaling differ between the two sexes: Differentiation and survival are triggered in granulosa cells, whereas proliferation and maturation are promoted in SCs. As a consequence, suppression of insulin/IGF signaling in granulosa cells leads to infertility, whereas suppression in SCs leads to males which, although able to produce offspring, show a dramatic reduction in their final pool of SCs.

### 5.4. The Role of the Insulin/IGF System in Steroidogenic Lineages: FLCs, ALCs and Adrenal Glands

Androgen production and fertility in sexually mature males are dependent on the postnatal differentiation of ALCs. ALCs originate from spindle-shaped progenitor Leydig cells (PLCs) around postnatal days 7–10 (P7–P10) [76,77]. These PLCs differentiate into round immature LCs between P10–20, then increase in size and number, and eventually mature into ALCs with enhanced steroidogenic capacity [76,77].

Testicular levels of IGF1 increase after P7 and peak at P24, coinciding with the beginning of pubertal rise in testosterone secretion and the timing of ALC differentiation and maturation [51]. In fact, IGF1 has been shown to act as a critical factor in the establishment of a normal number of LCs, their maturation and their steroidogenic capacity [50,78,79], both in vivo and in vitro. More recently, an in vivo deletion of *Insr* and *Igf1r* in steroidogenic cells showed that insulin/IGF signaling is required for adrenocortical development, as well for ALC maturation and steroidogenic function, but that it is dispensable for fetal LCs function [55]. As a result, mutant males show a 55% mortality rate due to adrenal defects, while the surviving males display quantitative alterations of sperm production, along with a failure to produce offspring because of an absence of mating behavior. Interestingly, cell-lineage tracing in this mouse model has allowed the discrimination between cell-autonomous and non-autonomous effects of insulin/IGF signaling in steroidogenic cells. Cell-autonomous steroidogenic failure in differentiated LCs was accompanied by non-cell autonomous PLC enrichment, suggesting the existence of a crosstalk within the steroidogenic lineage between cells at different steps of differentiation. Of note, the up-regulation of PLC markers observed in *Igf1* null mice could also be the result of an indirect PLC stimulation, rather than an arrest in the differentiation, as originally proposed by the authors [78].

It is worth noting here that the adrenal and testicular phenotypes resulting from *Insr* and *Igf1r* deletion in steroidogenic cells are similar to the dysgenesis observed in mice lacking the steroidogenic factor 1 gene (*Sf1*, officially *Nr5a1*) in this cell type [80]. Similar phenotypes between the two mouse models are also observed in the constitutive knockouts of these genes [54,81]. These evidence point toward a possible cooperation between NR5A1 and insulin/IGF signaling during several steps of adrenogonadal development. In fact, such direct cooperation has been shown in vitro in rat LCs [82]. In addition, the *Sf1*-mediated form of adrenal hypoplasia congenital syndrome in humans is characterized by primary adrenal failure and gonadal dysgenesis [83]. It is thus possible that insulin/IGF signaling is a conserved major regulator of steroidogenic lineage development and function, although a more thorough investigation is needed.

### 5.5. Cell-Autonomous Insulin/IGF Signaling Does Not Play an Essential Role in Germ Cell Development and Gamete Production

In vitro studies in several species have demonstrated a role for both insulin and IGF1 in oocyte maturation and early embryo development. For instance, the addition of IGF1 to the maturation medium of oocytes accelerates meiotic progression and increases the number of bovine blastocysts [84]. Similarly, the presence of insulin in bovine oocyte maturation medium leads to accelerated meiotic progression [85], but also exerts mitogenic and anti-apoptotic activities [86]. On the contrary, however, specific ablation of *Insr*, *Igf1r* or both receptors in mouse oocytes during follicle/oocyte growth and meiotic progression does not affect female fertility: Mutant females exhibit normal estrous cyclicity, oocyte development and maturation, parturition frequency and litter size [87], indicating that, at least in rodents, oocyte insulin/IGF signaling is dispensable for oocyte and follicular development, maturation, as well as early embryonic development.

Similarly, it has been shown that when either *Insr*, *Igf1r*, or both, are inactivated specifically in mouse male germ cells, spermatogenesis remains unaffected and adult individuals are fertile, with normal testis size, seminiferous epithelium histology, sperm production and reproductive functions [43].

This lack of phenotype in both male and female GC-specific mutants, along with the fact that other cell-specific mutants do show gonadal development and reproductive alterations, indicate that, at least in mice, IGFs may act primarily on somatic rather than germ cells.

## 6. Concluding Remarks and Future Perspectives

Over the last few decades, detailed and extensive studies have firmly established the essential role played by the insulin/IGF family in mammalian sexual development and reproduction. Mainly through the use of conditional knockout mouse models, several obstacles have been overcome and have led to significant findings. For instance, it is now clear that IGFs are absolutely essential for testis differentiation and function: more specifically, both receptors are necessary for testis specification [54]; for establishing SC number, testis size, and sperm output [43]; but also for LC development and steroidogenic function [55]. It is now also clear that insulin/IGF signaling plays an important role in the female reproductive function: IGF1 is essential for ovulation to occur normally [50], while granulosa cell-produced *Igf1r* is pivotal for steroidogenesis and follicle survival [75].

Obviously, although tremendous progress has been achieved over the years, there are several additional issues that need to be clarified:

First of all, the relative contribution of endocrine, paracrine and autocrine effects of IGFs in the testis and the ovary is still unclear. It is possible that some of the observed phenotypes result from a combination of such effects. Also, the eventual outcome of IGF activity on gonadal development and function could be further modulated by IGFBPs. Indeed, circulating and tissue resident IGFs can bind with differential affinity to the six IGFBPs, whose expression is cell type specific [7]. These interactions have been proposed to regulate IGF bioactivity both positively and negatively, depending on the tissue context [7]. Therefore—and given the importance of the insulin/IGF system for gonadal function—IGFBPs may represent another layer of regulation for sexual development and reproduction. It should be noted here that, although no gonadal phenotype has been reported in *Igfbp* null mice [88,89,90,91], dynamic *IGFBP* expression during folliculogenesis has been observed in humans, thereby suggesting a relevant role for these genes in female fertility [90].

Second, although numerous knockout animal models have been created, we still do not have an in vivo conditional model for every member of the insulin/IGF family in each cell type of the testis and the ovary. Such a “collection” of animals would most certainly provide us with a more complete understanding of the precise intratesticular and intraovarian roles played by insulin/IGF family members.

Third, the exact mechanism through which insulin/IGF signaling functions in the male and female reproductive organs is still not fully understood. Very few molecular mechanisms have been proposed in order to explain the phenotypes observed in the animal models that have been described in this review. For instance, the reduced testis size observed in SC-*Insr;Igf1r* male mice might be attributed to the reduction of *Nr5a1* expression: Baba et al. have shown that suppression of *Nr5a1* by siRNA reduces the production of ATP and NADPH, but also lowers the expression of genes involved in glucose metabolism [91]. One might hence postulate that disruption of the *Nr5a1* gene in vivo could lead to insufficient production of ATP and NADPH through impaired activation of glucose metabolism, and thereby result in dysgenesis of steroidogenic tissues [91]. Similar molecular explanations for other knockout phenotypes are needed in order to fully comprehend how insulin/IGF signaling functions in testes and ovaries.

Lastly, another aspect that requires further investigation is whether insulin/IGF signaling acts in coordination—or in antagonism—with other signaling pathways within the reproductive organs. For example, since both IGF1R and FSHR activate AKT signaling [92], and since studies in male as well as in female mice [43,75] have revealed that insulin/IGF signaling is actually crucial for FSH in order to mediate its proliferative and differentiation effects in Sertoli and granulosa cells, respectively, it has been proposed that a crosstalk between the two pathways exists. Unraveling the precise “mechanistic” details of this crosstalk would be of great importance in the context of human infertility treatment [62]. Therefore, a careful evaluation of all signaling pathways that are active during sexual development and reproduction is much needed, as it will undoubtedly offer interesting new perspectives in reproductive biology.

Fortunately, novel technologies that have been developed over the past few years currently provide researchers in the field with extremely powerful tools. The possibility of individual cell profiling at the transcriptome, proteome—or even epigenome—level will certainly prove to be an instrument of outstanding significance: knowing the exact molecular profile of each cell type in the testis and the ovary, at different stages of development, will help us comprehend in depth the mechanisms that control sexual development. From a medical perspective, such knowledge will offer important insight with regards to human infertility treatment. Moreover, if performed in parallel in multiple species, such analyses could yield significant information about the evolutionary history of reproductive function.

## Figures and Tables

**Table 1:** Summary of insulin/IGF system expression pattern in developing testis, mouse models and human phenotypes related to sexual development and reproduction.

			*Ins2/INS*	*Igf1/IGF1*	*Igf2/IGF2*	*Insr/INSR*	*Igf1r/IGF1R*	*Irs1/IRS1*	*Irs2/IRS2*
Mouse XY	Gene expression ^1^	Interstitial progenitors	-	+++	+++	-	+++	-	+
		Leydig cells	-	+	+	+	+++	-	+
		Sertoli cells	-	-	-	-	+++	-	+++
		Germ cells	-	-	+	-	+++	-	+
		Endothelial cells	-	+	+++	-	+++	-	-
	Phenotypes		- Insulin-injected and HFD mouse models: reduced steroidogenic enzyme gene expression and steroidogenesis [49]	- Constitutive KO: steroidogenic failure, PLC markers upregulation, reduced testis size and sperm count [50,51]	- Constitutive KO: no testicular defects [52]	- Constitutive double KO: reduced proliferation rates of somatic progenitors, male-to-female sex reversal [53,54]		- Constitutive KO: no testicular defects [44]	- Constitutive KO: reduced testis size and sperm count [44]
						- LC specific-KO: adult Leydig cells (ALCs) maturation defects, cell-autonomous steroidogenic failure, non-cell autonomous PLCs (progenitor Leydig cells) enrichment, reduced testis size and sperm count. Fetal LCs (FLCs) not affected [55]			
			- LC specific-KI: age-dependent germ cell degeneration [56]			- Sertoli (SC) specific-KO: reduced follicle stimulating hormone (FSH)-dependant SC proliferation, testis size and sperm count [43] acting through IGF/PTEN/PI3K pathway [42]			
						- GC specific-KO: no testicular defects [43]			
Human XY	Gene expression ^1^	Interstitial progenitors	-	+++	+	+++	+++	-	-
		Leydig cells	-	-	-	+++	+++	+++	+++
		Sertoli cells	-	-	-	+++	+++	-	-
		Germ cells	-	+	-	+	+++	-	-
		Endothelial cells	-	-	-	+++	+++	-	-
	Conditions			- Homozygous mutation: reproductive system not affected [57,58,59,60]	- Paternally inherited *IGF2* mutation: ambiguous genitalia, penoscrotal hypospadia, unilateral cryptorchidism, hypogonadism [61]				
				- Positive correlation between IGF1 and testicular volume [62]					
				- IGF1 administration to Laron syndrome patients: increased testis size [62]					

^1^ Single-cell gene expression from [42] and [46]. Abbreviations: -, barely expressed; +, moderately expressed; +++, robustly expressed; HFD, high-fat diet; KI, knock-in; KO: knock-out.

## Abbreviations

AKT	Protein kinase B
ALC	Adult Leydig cell
ATP	Adenosine triphosphate
CTNNB1	Catenin (cadherin associated protein), beta 1
CYP19A1	Cytochrome P450, family 19, subfamily a, polypeptide 1
E	Embryonic day
ERK	Extracellular signal-regulated kinase
FGF9	Fibroblast growth factor 9
FLC	Fetal Leydig cell
FOXL2	Forkhead box L2
FSH	Follicle stimulating hormone
HCG	Human chorionic gonadotropin
HPG	Hypothalamic–pituitary–gonadal
GC	Germ cell
GH	Growth hormone
IGFs	Insulin-like growth factors
IGF1R	Insulin-like growth factor 1 receptor
IGF2R	Insulin-like growth factor 2 receptor
IGFBP	Insulin-like growth factor binding protein
INS	Insulin
INSR	Insulin receptor
INSR-A	Insulin receptor isoform A
INSR-B	Insulin receptor isoform B
IRR	Insulin receptor-related receptor
IRS	Insulin receptor substrate
JAK	Janus-family tyrosine kinase
LC	Leydig cell
M6P/IGF2R	Mannose-6-phosphate/IGF2 receptor
MAPK	Mitogen-activated protein kinase
NADPH	Nicotinamide adenine dinucleotide phosphate
NR0B1	Nuclear receptor subfamily 0 group B member 1 (also known as DAX1)
NR5A1	Nuclear receptor subfamily 5, group A, member 1 (also known as Sf1)
P	Postnatal day
PGC	Primordial germ cell
PI3K	Phosphatidylinositol-3 kinase
PIP2	Phosphatidylinositol 4,5-bisphosphate
PIP3	Phosphatidylinositol 3,4,5-trisphosphate
PLC	Progenitor Leydig cell
PTEN	Phosphatase and tensin homolog deleted on chromosome 10
RA	Retinoic acid
RAF	Rapidly accelerated fibrosarcoma
RSPO1	R-spondin 1
SC	Sertoli cell
SHC	Src homology domain-containing
siRNA	Small interfering RNA
SOX9	SRY (sex determining region Y)-box 9
SRY	Sex determining region of chromosome Y
STAT	Signal transducer and activator of transcription
TPM	Transcripts per million
WNT4	Wingless-type MMTV integration site family, member 4
